# Determinants of Ukrainian Mothers’ Intentions to Vaccinate Their Children in Poland: A Cross-Sectional Study

**DOI:** 10.3390/vaccines13030325

**Published:** 2025-03-19

**Authors:** Katarzyna Lewtak, Joanna Mazur, Harriet Dwyer, Agnieszka Sochoń-Latuszek, Anastasiya Atif, Tomasz Maciejewski, Dorota Kleszczewska

**Affiliations:** 1 Department of Social Medicine and Public Health, Medical University of Warsaw, 02-106 Warsaw, Poland; 2 Department of Humanization of Medicine and Sexology, Collegium Medicum, University of Zielona Góra, 65-046 Zielona Góra, Poland; j.mazur@inz.uz.zgora.pl; 3 Department of Global Health and Development, London School of Hygiene and Tropical Medicine, London WC1E 7HT, UK; harriet.dwyer@lshtm.ac.uk; 4 UNICEF Refugee Response Office in Poland, 00-869 Warsaw, Poland; asochon@unicef.org (A.S.-L.); aatif@unicef.org (A.A.); 5 Clinic of Obstetrics and Gynaecology, Institute of Mother and Child, 01-211 Warsaw, Poland; tomasz.maciejewski@imid.med.pl; 6 Institute of Mother and Child Foundation, 01-211 Warsaw, Poland; dorota.kleszczewska@imid.med.pl

**Keywords:** vaccination intention, vaccine uptake, national immunisation programme, vaccine hesitancy, refugees, Ukraine, Poland

## Abstract

Background/Objectives: In 2022, the full-scale invasion in Ukraine forced over 6 million Ukrainians, primarily mothers and children, to seek safety outside of the country. This massive influx has posed a significant challenge to the Polish healthcare system, particularly regarding routine vaccination for children. This study aims to examine the vaccination intentions of displaced Ukrainian mothers, their compliance with the Polish National Immunisation Programme (PNIP), and the factors that influence these intentions. Methods: A web-based survey (June–July 2023) was conducted among Ukrainian mothers in Poland. The questionnaire assessed the importance placed on vaccination, knowledge of PNIP, and concerns related to displacement and vaccination. Hierarchical logistic regression identified key determinants. Results: Among 2572 respondents, 64.5% reported that their children had received only some or none of the recommended vaccines. Key barriers included unfamiliarity with PNIP, limited knowledge of vaccines, and concerns about vaccine side effects. Of mothers whose children had not followed PNIP, 41.7% intended to vaccinate, 33.1% refused, and 25.2% were undecided. Regression analysis identified perception of vaccination importance as the strongest predictor. Partial adherence to PNIP doubled vaccination likelihood, while a firm plan to return to Ukraine reduced it 2.4 times. Mistrust in vaccines increased refusal risk tenfold. The final model confirmed mothers’ attitudes towards vaccination and future plans (return to Ukraine) as dominant factors. Conclusions: This study underscores the complex determinants shaping vaccination decisions in conflict-displaced communities. It provides insights for public health strategies to enhance vaccine uptake by reducing access barriers, restoring trust, and strengthening vaccine literacy.

## 1. Introduction

Vaccination coverage, particularly for routine immunisation, constitutes one of the fundamental pillars of preventive healthcare in any society [1]. Recently, the world has faced a critical health crisis affecting childhood vaccination after backsliding in vaccine coverage during the COVID-19 pandemic; leaving millions of children vulnerable to deadly vaccine-preventable diseases [2]. Inequity in health systems has meant that children from marginalised communities have historically had lower vaccination rates. Sustained action is required to prevent further backsliding on immunisation efforts with a particular focus on reaching the hardest to reach children [3].

Vaccination services are critical for populations who have been displaced [4,5]. A study by Nakken and colleagues, using data extracted from the Danish Red Cross database, for example, revealed that nearly one-third of children and adolescents seeking asylum in Denmark required additional vaccinations due to gaps in their immunisation coverage [6]. In 2022, the full-scale invasion in Ukraine caused mass displacement, with Poland becoming a primary destination for families seeking refuge [7]. This massive influx has posed challenges for the Polish health system, including in the adequate provision of routine vaccination services for Ukrainian children. This was important as Ukrainian children and caregivers were integrated into Polish communities including through access to nurseries, kindergartens, and schools. Prior to the escalation of war in 2022, Ukraine already had some of the lowest vaccination rates in Europe. Multiple factors have led to the low vaccination rates in Ukraine, including widespread vaccine hesitancy, which has been exacerbated by social media campaigns spreading mis- and disinformation and eroding public confidence in Ukrainian authorities [8].

Vaccine hesitancy is defined as ‘a behaviour, influenced by a number of factors including issues of confidence (level of trust in vaccine or provider), complacency (do not perceive a need for a vaccine, do not value the vaccine), and convenience (access)’, known as the 3Cs model, updated in 2018 to the 5Cs, replacing convenience with constraint and adding calculation and collective responsibility [9,10]. Vaccine hesitancy is a complex issue influenced by multiple factors, including personal motivation, lack of knowledge, doubts about the benefits of vaccination, overconfidence in one’s ability to avoid illness, anxiety about vaccines, and fear of side effects, among others. From historical mistrust in medical institutions to the widespread dissemination of mis- and disinformation through social media, the drivers of vaccine hesitancy are complex and require tailored approaches to effectively address [11]. While vaccine hesitancy has been widely studied, existing research largely focuses on general predictors such as concerns about vaccine safety, misinformation, and distrust in healthcare systems. However, displaced populations, including Ukrainian refugees, face additional, unique barriers. These include challenges in navigating an unfamiliar healthcare system, uncertainty about long-term residence status, language barriers, and the psychological burden of forced migration. Understanding how these factors interact with known predictors of vaccine hesitancy is crucial for designing targeted interventions that improve vaccine uptake in this specific group [12,13,14].

To address the vaccination needs of refugees from Ukraine, European and international agencies have developed guidelines and protocols for host countries. These documents recommend ensuring access to vaccines for refugees and emphasize the importance of closing existing immunisation gaps and improving vaccination coverage in the host population as well. The main priority was to ensure that refugees receive at least age-appropriate doses of vaccines against poliomyelitis, measles, rubella, and COVID-19, preferably by integrating them into the national immunisation programme of the host countries, following a life-course approach [15,16,17]. Displaced Ukrainians were also granted open access to Polish health insurance, which enabled access to Polish healthcare [18]. Despite various efforts, vaccination rates among Ukrainian children in Poland remain below optimal levels [19].

In Poland, according to national law, all children and adolescents under the age of 19 who have been residing in the country for more than three months are required to be vaccinated, in line with the annually updated national vaccination schedule [20]. For Ukrainians displaced in Poland this has meant navigating the Polish National Immunisation Programme (PNIP), and vaccinations included in the PNIP were offered to children from Ukraine free of charge. In responding to the needs of displaced Ukrainians and host communities in Poland, there has been an imperative to strengthen vaccination services as a critical aspect of integration into the Polish health system. Despite this, there remains a knowledge gap around the preventative health behaviours of displaced Ukrainians. Building the evidence base around the health behaviours of displaced Ukrainian mothers, including decision-making processes and their understanding of vaccines, will provide valuable insights for public health practitioners and policymakers working to increase the uptake of routine vaccination across the region. This study is a continuation of the author’s interest in analysing the drivers of vaccine hesitancy [12,19]. Despite previous research, and existing international literature, there is still limited information about the factors influencing the attitudes of displaced mothers towards vaccination and their practices around childhood vaccination in their host country. Existing research highlights the impact of context on drivers of vaccine hesitancy and barriers to vaccination uptake. Deeper understanding of these factors amongst displaced Ukrainians in Poland is helpful to inform efforts aimed at promoting childhood vaccination and improving vaccination coverage rates [13,21].

This study aims to examine the vaccination intentions of displaced Ukrainian mothers, their compliance with the Polish National Immunisation Programme, and the factors that influence these intentions.

## 2. Materials and Methods

### 2.1. Study Design and Participants

A quantitative web-based study was conducted online between 27 June and 18 July 2023. It targeted adult Ukrainian mothers who had fled the country and been residing in Poland since the beginning of the full-scale war. Participant recruitment was managed by market research company, Rating Online, based in Kyiv, Ukraine. Recruitment was carried out through social media advertisements and text messages sent to phone numbers from mobile providers Kyivstar and Vodafone. For data protection purposes, the providers sent the messages, and the research team did not have access to the phone numbers. Mobile providers were able to identify individuals who had been in Poland for at least 30 days since the start of the year and whose last contact in Poland occurred after their last contact in Ukraine. To ensure the correct group was reached, screening questions at the start of the survey confirmed the participant’s current residence, age, nationality, sex, and whether they had at least one child under 7 years of age. The survey was anonymous.

Before starting the survey, participants reviewed an information sheet outlining the study’s purpose and were asked if they wished to participate. The study commenced only after informed consent was obtained. It was available in both Ukrainian and Russian, and participants selected their preferred language.

**Figure 1:**
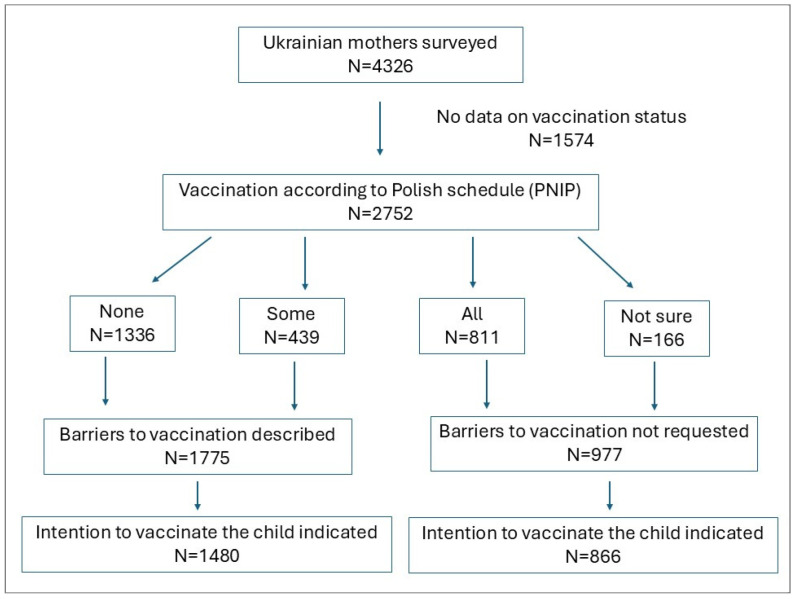
Flowchart of sample design: highlighting variability in group sizes taken for further analysis.

Figure 1 shows the flowchart of sample design: highlighting variability in group sizes taken for further analysis.

The questionnaire was partly adapted from the published behavioural and social drivers of vaccination (BeSD) questionnaire, including questions on the perceived importance of vaccination, vaccination intentions, social norms, trust in healthcare providers, and knowledge of the vaccination schedule [22]. It also featured items tailored to the context of Ukrainian displacement in Poland. This included possession of a vaccination card and the likelihood of returning to Ukraine. The complete questionnaire can be found in the Supplementary Materials.

The study was conducted according to the guidelines of the Declaration of Helsinki and approved by the Bioethics Committee at the Institute of Mother and Child in Poland (number 33/2023 on 28 April 2023).

### 2.2. Measures

Socio-demographic factors included age of Ukrainian mothers, education level (categorized as Primary, Secondary, Vocational, Incomplete Higher, or Higher/Scientific degree), Polish language proficiency (assessed as High, Medium, Low, Do not speak Polish at all, or Prefer not to say), intention to return to Ukraine (categorized as “As soon as possible”, “Probably return soon”, “Probably not return even if possible”, “Will not return in any case”, and “Not sure”), and the age of their youngest child (grouped into three categories: 0–2 years, 3–4 years, and 5–7 years).

The vaccination status of the child (compliance with Polish National Immunisation Programme) was measured by asking mothers whether their child had received none, some, or all vaccines recommended in the Polish National Immunisation Programme, with an additional option for those unsure or unable to answer.

Intentions to vaccinate the child in host country according to the National Immunisation Programme were based on one item (“Do you intend to vaccinate your child in the next 6 months in Poland?”) with response options: “yes”, “no”, and “difficult to answer/not sure”. In this question, we considered each vaccination that was recommended in PNIP for a child at a given age, as well as catch-up vaccinations to fill gaps in the immunisation history, without specifying a particular vaccine and/or vaccination. In the question regarding the intention to vaccinate a child in Poland, a six-month time perspective was adopted due to the fact that since the escalation of war in Ukraine in February 2022, the immunisation system was heavily impacted. Many children were not able to access vaccinations in accordance with the Ukrainian Immunisation Programme recommended for their age, and this timeframe provides a snapshot of the vaccination intentions during one point in the displacement experience of mothers. Official statistical data on child vaccination rates indicated the need to catch up on missing vaccinations. Additionally, this timeframe was chosen to assess the scale of vaccine hesitancy among Ukrainian mothers and their reluctance to make decisions about vaccinating their child in the host country in order to plan and implement interventions to increase vaccine uptake among Ukrainian children.

Perceived importance of vaccinating one’s child was measured using the item: “I think it’s important to vaccinate my child”. Response options were presented on a 10-point Likert scale ranging from “0—I strongly disagree” to “10—I strongly agree”. An ordinal variable was coded as follows: low importance (responses of 0–6), average importance (7–8), and high importance (9–10).

Reasons for not vaccinating their child, if some or none of the vaccines were received, were assessed using multiple response options. The item asked: “What was the reason for your decision not to vaccinate your child?” The possible responses included (a) I’m concerned about the safety and side effects of vaccines, (b) I don’t have easy access to healthcare services in Poland, (c) I don’t know the vaccine schedule/requirements in Poland, (d) I don’t know where to get vaccinated in Poland, (e) I don’t trust the healthcare system and/or medical authorities in Poland, (f) I don’t believe my child needs to be vaccinated (don’t believe in the threat of the diseases), (g) I was advised by a health provider not to receive the vaccines (contraindications, etc.), (h) I refuse to vaccinate my child due to my religious beliefs, (i) I prefer to vaccinate my child when back in Ukraine, (j) Other (please, specify), (k) Difficult to answer/Not sure.

### 2.3. Statistical Analysis

Categorical variables were presented as numbers and percentages. Their distributions were compared using the chi-square test in groups distinguished by adherence to the Polish vaccination calendar and intention to vaccinate the child later.

When concluding on the differences between groups of mothers from the contingency tables, the value of standardised adjusted residuals was taken into account, and their absolute value above 2 demonstrated significant differences in a given group compared to the others.

A hierarchical multiple logistic regression model was used to examine the determinants of intention to vaccinate a child in Poland in the next 6 months. The dependent variable took the value 1 if the mother wanted to vaccinate her child and 0 if she did not want to or was unsure of her decision. Three hierarchical levels were applied, grouping the independent variables into three blocks. Block 1 contained only the mother’s basic data and the child’s age. In the second block, reasons for previous non-adherence to the Polish vaccination calendar were introduced, coded as zero-one variables. In the last block, the analysis was adjusted for the mother’s perceived level of importance of having her child vaccinated. Only this variable and the child’s age were treated as continuous variables. Variables were entered into models according to Wald’s stepwise selection method.

Results were presented as odds ratios (OR) with 95% confidence interval (CI) and the significance level of the regression parameter according to the Wald test. To evaluate the goodness of fit of the logistic regression models, Nagelkerke’s pseudo-R squared statistics was calculated. Due to the critical importance of estimating the association with reasons for non-vaccination, all models were estimated on a sample of mothers who indicated these reasons (level of compliance with Polish vaccination schedule none or some).

As a complementary analysis, the structure of reasons for not vaccinating a child was checked using principal component analysis (PCA), with varimax rotation, and the results are included in the appendix. SPSS version 29.0 (IBM Corp, Armonk, NY, USA) software was used for statistical analyses, and a *p*-value < 0.05 was considered significant.

## 3. Results

Among 2752 Ukrainian mothers who provided information on their children’s vaccinations according to the Polish National Immunisation Programme, 1775 (64.5%) reported that their children had received only some or none of the recommended vaccines (non-compliance to Polish Immunisation Programme). These mothers identified perceived barriers to vaccination, and among them, 1480 (83.4%) expressed an intention to vaccinate their child within the next six months in Poland.

Table 1 presents the characteristics of the analysed group of 2752 Ukrainian mothers. Their mean age was 34.26 years (SD = 5.7). These were mothers of at least one child aged up to 7 years. The average age of the youngest child was 3.6 years (SD = 2.2). Over two-thirds of the mothers had education above the secondary level, and their proficiency in the Polish language could be described as average. One-quarter of respondents considered staying in Poland, although a significant proportion were unable to specify their intentions in this regard. These women considered vaccination of their child to be important, with 61.8% rating its importance at a minimum of 9 points on a scale from 0 to 10. The average importance score for vaccination was 7.67 points (SD = 3.37). Mothers of children partially vaccinated according to the PNIP, compared to mothers of non-vaccinated children, were significantly more likely to have a higher level of education (education above vocational: 67.2% vs. 63.2%, *p* = 0.011), less likely to report not speaking Polish (5.2% vs. 9.5%, *p* < 0.001), and plan a return to Ukraine (37.0% vs. 43.2%, *p* = 0.002), and more inclined to consider child vaccination to be of high importance (64.7% vs. 52.5%, *p* < 0.001).

**Figure 2:**
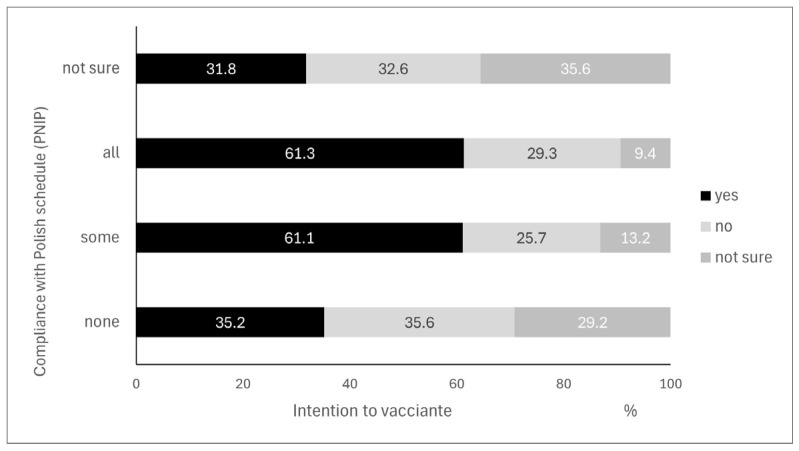
Intention to vaccinate the child within the next 6 months in Poland, based on the current vaccination status (N = 2346) (figure to be included).

In the surveyed sample of 2752 mothers, 40.3% were determined to vaccinate their child within the next six months. A significant association was found between the vaccination status of children and these intentions (*p* < 0.001). The percentage of mothers not planning to vaccinate their child in Poland was highest when the vaccination schedule according to the Polish scheme for children of a given age was not followed at all. Additionally, an inability to answer this question was also a burdening factor. No significant difference in intentions was observed between fully and partially adhering to the Polish vaccination schedule (*p* = 0.103). However, attention can be drawn to the elevated percentage of mothers uncertain about their intentions if their child had not been fully vaccinated (Figure 2).

Attention was also given to the characteristics of mothers who were uncertain about their intentions regarding vaccinating their child in Poland. Compared to mothers who had a clear stance (either in favour or against vaccination), uncertain mothers were found to have (a) a higher proportion with a secondary level of education (16.6%), (b) much lower proportion of those with young children under the age of two (17.6%), (c) a lower proportion of those who spoke Polish fluently (2.5%), and (d) greater uncertainty regarding their potential return to Ukraine (46.2%). In comparison, mothers who decided not to vaccinate their child in Poland in the near future: (a) were much less likely to describe their education as incomplete higher (9.1%), (b) were more likely not to speak the Polish language at all (10.4%), (c) were much more likely to decide to return to Ukraine quickly (27.1%) and (d) their assessment of the importance of vaccination was more likely to be low, even much lower than in mothers not sure about their intention (42.0%). Mothers opposed to vaccination in Poland, similarly to undecided mothers, were less likely to have very young children under two years of age (24.3%). Detailed data comparing the three groups of mothers are provided as Supplementary Materials (Table S1).

Table 2 summarizes the reasons for not vaccinating children as reported by 1775 mothers who indicated that they had not previously adhered to the Polish vaccination schedule (responses “none” and “some”). The most frequently cited reasons were a lack of knowledge about the regulations in Poland and concerns about side effects. The responses “other” and “difficult to answer” were also commonly chosen and could co-occur with specific reasons listed in the survey. Ukrainian refugee mothers usually reported between one and three reasons for not vaccinating their children in Poland, with a maximum of six reasons provided. Of the mothers surveyed, 68.7% provided exactly one reason, 19.3% reported two reasons, and 8.7% indicated three reasons. The two most commonly reported reasons, which appeared together (in Table 2), were “I don’t know the vaccine schedule/requirements in Poland” and “I don’t know where to get vaccinated in Poland”. These reasons were mentioned by 14.8% of respondents who provided explanations. The co-occurrence of other pairs of reasons was much less common, with a maximum of 4.4% of recorded cases.

As shown in Table S2 provided in the Supplementary Materials, three groups of reasons can be identified: those related to unfamiliarity with the Polish healthcare system, safety concerns, and reliance on the Ukrainian healthcare system. The ranking of reasons varied depending on the child’s vaccination status. In cases of partial vaccination, undefined reasons ranked first, followed by safety concerns regarding potential side effects among the specified reasons. Vaccination status influenced the frequency of reporting five specific reasons, which were more common when the child had not been vaccinated at all.

In the group of 1480 Ukrainian mothers whose children had not followed the Polish vaccination schedule, an analysis was conducted to determine how specific reasons for previous withdrawal from vaccinations influenced their subsequent intentions to vaccinate (Table 3). Overall, 41.7% of this group expressed a willingness to vaccinate their child, 33.1% had no such intention, and the remaining 25.2% were undecided. The data presented in Table 4 allow this general distribution to be compared with cases where a specific reason for earlier non-vaccination was indicated.

Analysing groups of factors according to the classification provided in Table S1, it can be observed that mothers unfamiliar with the Polish healthcare system were more likely to express the intention to vaccinate their child. Similarly, mothers who cited a variety of reasons (responses “other” and “not sure”) were often inclined to vaccinate their child. A lack of willingness to vaccinate was particularly common when earlier non-vaccination was attributed to general distrust of vaccines or reliance on the Ukrainian healthcare system. Mothers who cited religious reasons were the least likely to vaccinate their children; however, this reason was rarely reported overall.

In Table 4, the results of three hierarchical logistic regression models are presented.

In the first model, significant factors included the child’s age, prior compliance with the Polish vaccination schedule, and the mother’s expressed intention to return to Ukraine. Vaccination status was a factor introduced in the first step, where partial compliance with the Polish vaccination schedule doubled the chances of later vaccinations. The older the child, the lower the likelihood that the mother would plan to vaccinate them in Poland. If the mother was determined to return to Ukraine as soon as possible, the intention to vaccinate the child in Poland decreased by 2.4 times compared to those who decided to stay in Poland. Hesitation about returning to the country (the two middle response categories) no longer influenced vaccination intentions. The level of proficiency in the Polish language and the mother’s education were not qualified for the first model. The three factors qualified in the first model remained significant in the two subsequent models.

In the second model, eight factors were included, which were indicated as reasons for non-compliance with the Polish vaccination schedule. The responses “others” and “difficult to say”, as well as sporadically mentioned religious reasons, were excluded from this group. Including reasons for the child’s prior non-vaccination significantly improved the model’s fit, measured by the pseudo-R^2^ coefficient. One of the qualified reasons increased the likelihood of planning to vaccinate the child, which was the lack of knowledge about where vaccinations could be performed. The other four reasons significantly influenced the reluctance to later vaccinate the child, with the most significant factor being a lack of belief that vaccination was necessary for the child. For this attitude, the risk of opting out of further vaccinations in Poland increased tenfold. However, adherence to the Polish vaccination schedule remained the dominant predictor introduced in the first step.

In the final model, the mother’s opinion on the importance of vaccinating the child was included. This factor proved to be the most significant and replaced two earlier reasons for not reporting for vaccinations. The second factor introduced to the model was the intention to return to Ukraine. The unqualified reasons identified earlier in the second model represented a general distrust of vaccinations. The third model had a better fit than the second model.

## 4. Discussion

Vaccination, widely recognized as one of the most effective public health strategies, has encountered growing hesitancy, often linked to factors such as risk perception, trust in government and healthcare systems, past vaccine experiences, misinformation, concerns about side effects, and political beliefs [14,23].

The sudden influx of displaced Ukrainians to Poland in 2022 created numerous challenges for the healthcare system. These primarily concerned the epidemiological situation and the risk of spreading infectious diseases, including COVID-19 and other vaccine-preventable diseases [24]. The challenges for the healthcare system also involved ensuring migrants’ access to healthcare in the host country, at a time when the healthcare system’s resources were severely affected by the impact of the COVID-19 pandemic. The full-scale Ukraine war has made it more difficult for some Ukrainians to get vaccinated. Key obstacles include the displacement of populations, the destruction of healthcare infrastructure, and the disruption of logistical routes for the delivery of medical supplies. Additionally, many children born during the pandemic missed routine vaccinations, making this cohort of children particularly susceptible and in need of catch-up vaccinations. Due to low vaccine coverage, Ukraine has recently faced outbreaks of vaccine-preventable diseases (i.e., polio, measles) [25]. The risk of an epidemic in newly arrived refugees is influenced by their country of origin and the epidemiological situation in the region at which they are arriving [26,27]. In countries hosting displaced Ukrainians, ensuring health security has become a priority, both for this vulnerable group and for the local communities [28]. In response to this crisis, innovative strategies should be implemented to strengthen access to immunisation and to mitigate the long-term public health consequences and gaps in immunisation coverage.

The WHO’s Immunization Agenda 2030 (IA2030), and subsequent reports, emphasize the importance of ensuring equitable vaccine access for marginalized groups, including migrants and refugees, and highlights the need to incorporate catch-up vaccinations for missed doses across the life course [22,29,30]. WHO underscores the principle that “it’s better to vaccinate late than never”. It encourages countries to recognize barriers and drivers of vaccine uptake in accordance with National Immunisation Programmes and establish clear policies and schedules for catch-up vaccination to address immunisation gaps that widen with age [31]. Our study elucidates the complex dynamics that shape a mother’s intention to vaccinate and how they sit within the wider experience of displacement. The WHO Strategic Advisory Group Experts (SAGE) on Immunisation has defined three key domains of influence impacting vaccine hesitancy including (1) confidence (trust in the safety or efficacy of the vaccine), (2) complacency (perception of the risk of disease and importance of immunisation), and (3) convenience (ease of access) [32,33]. The findings in this research demonstrate these three key domains of influence at play and provide a helpful framework for consideration of the results.

### 4.1. Confidence: Vaccine Safety and Trust

Confidence refers to the level of trust in a vaccine’s effectiveness and safety, the reliability and competence of the healthcare system and professionals administering vaccines, and the intentions of policymakers working to ensure vaccine accessibility. A lack of confidence often stems from strongly negative attitudes towards vaccination, which can be shaped by misinformation about vaccine risks, membership in anti-vaccine groups, or legitimate concerns about vaccine safety and efficacy [32,34].

In terms of confidence, higher levels of trust were associated with higher acceptance or intention to vaccinate. This relationship was observed for trust in the vaccine itself, science, healthcare providers, government institutions, and media sources [35].

Nearly a quarter of mothers expressed concerns about vaccine safety (21.5%). This lack of confidence in vaccines decreased the likelihood to vaccinate in the next six months. A smaller proportion of respondents cited a lack of trust in the Polish healthcare system (6.1%). These findings align with the idea that trust, including in the safety of vaccines and the wider health system, is a crucial factor in vaccine decision making [36].

In studies conducted by Gańczak et al., Ukrainian migrants and war refugees reported accepting and administering mandatory child vaccines according to the Polish vaccination schedule and generally believed that vaccines given in Poland are safe and of high quality. When asked about the reasons for not participating in vaccinations, they cited mistrust in the government, health authorities, and services, which led to uncertainty about vaccine safety, quality, and effectiveness. Three types of mistrust were identified: (1) concerns about the competence and integrity of government authorities in recommending and providing vaccines, as well as ensuring adequate training for medical professionals; (2) mistrust was directed at local vaccination providers regarding their qualifications, potential corruption, and vaccine safety; and (3) trust in the vaccine itself was undermined, with doubts about the ability of manufacturers to deliver effective and safe products [37].

As research findings indicate, vaccine confidence is not merely an individual matter, but also a social and political issue. While the question of how much confidence is sufficient to support vaccination remains unanswered, it is clear that higher confidence in immunisation programmes is linked to lower vaccine hesitancy. It supports the idea that confidence in vaccination is tied to trust in the broader system with which it is connected [33].

It is important to consider the broader context when discussing trust. Trust is not formed in a vacuum but shaped by experience [38,39]. This could include past experiences with vaccinations in Ukraine, experiences within the Polish health system, and the cultural, economic and social factors that have evolved through the displacement journey. It is, therefore, important to not solely focus on individual attitudes to vaccines but consider the wider context. Building trust in vaccinations and overcoming vaccine hesitancy in the context of displacement crises relates to the following key areas: (1) Engaging displaced communities to understand the drivers of low confidence and investing in integrating these communities into wider host communities where vaccination confidence is higher, shaping social norms in the process. (2) Effectively managing unexpected events such as changes in the protected status of Ukrainians in Poland to ensure continued access to health services. (3) Enhancing the quality of the vaccination experience and interactions between individuals and their health system. (4) Using social data to help governments make informed decisions based on social experiences as well as biomedical insights while increasing their ability to communicate adequately and transparently with the communities that they serve [40].

### 4.2. Complacency: A Mother’s Own Beliefs

Complacency reflects the extent to which individuals perceive vaccination as necessary for preventing vaccine-preventable diseases. It arises from a combination of factors, including risk perception, knowledge of the disease and vaccine, concerns about side effects or other reactions, and beliefs about the necessity of vaccination. Complacency can be shaped by the prioritization of health relative to other responsibilities and may paradoxically result from a decreased perception of risk due to the success of immunisation programmes. Additionally, self-efficacy—an individual’s perceived or actual ability to take action and vaccinate—plays a crucial role in determining the influence of complacency on vaccine hesitancy [32].

Vaccination complacency exists where perceived risks of vaccine-preventable diseases are low, and vaccination is not deemed a necessary preventative action [9]. The study’s logistic regression model confirms this, finding that, among mothers who had not complied with the Polish National Immunisation Programme, the most significant predictor of intention to vaccinate was the importance they placed on vaccination.

Before the escalation of war in 2022, Ukraine had some of the lowest vaccination rates in Europe, mainly due to a high level of vaccination scepticism, online vaccine misinformation campaigns, and structural failures in the Ukrainian health system. The coverage of childhood immunisation consistently remained below the WHO-recommended thresholds necessary to achieve herd immunity against several severe diseases. According to data from the Public Health Centre of the Ministry of Health of Ukraine, in 2022, only 69.1% of children in Ukraine received full vaccination against measles (2nd dose of MMR vaccine), while 68.9% were vaccinated against poliovirus in their first year of life. The ongoing conflict has caused mass displacement and seen the large-scale damage of health infrastructure, which may have further impacted immunisation rates [41]. In Poland, vaccination coverage against diseases targeted by the Polish National Immunisation Programmes (PNIP) remains at a level that ensures herd immunity, preventing epidemic outbreaks. In 2022, 94.1% of children in their second year of life were vaccinated against poliomyelitis, while 90.9% received the first dose of the MMR vaccine against measles. Mandatory vaccinations included in the PNIP are widely accepted by Poles, ensuring a high vaccination rate [26]. Results of a systematic review by Lee et al. revealed that the introduction of mandatory vaccination increases vaccination coverage rates [42]. A legal obligation to vaccinate a child, as well as belief that vaccination is right and is an effective way of disease prevention, and free-of-charge mandatory vaccinations were examples of parental motivators in the scoping review conducted in Poland by Szałast et al. [43].

In a survey conducted by Cholewik et al. in Poland during the first year of the Ukrainian war, 9.87% of parents reported that the influx of displaced Ukrainians influenced their decision to vaccinate their children. This group showed greater awareness of the differences in infectious disease epidemiology between Poland and Ukraine and had a more positive attitude towards recommended vaccinations [44].

However, the study also found that some mothers, even with prior hesitations, still intended to vaccinate. This demonstrates the complexity of vaccination dynamics. A study on messaging prompts for Ukrainian mothers in Poland, shows that messaging around risk aversion performed well [19]. An acknowledgement of the challenges faced by Ukrainian refugees and the expression of empathy could be an effective approach for helping address risk perception and complacency.

### 4.3. Convenience: The Experience of Displacement

Vaccine convenience is determined by factors such as its availability, affordability, willingness to pay, geographical accessibility, comprehension and acceptance of vaccine-related information (including language, cultural, and health literacy considerations), the attractiveness of immunisation services, and the quality of care provided [32].

Conflict and displacement often cause a disruption to the information flow used to guide individual health decisions [45,46]. Ukrainian mothers in Poland have had to navigate new vaccination schedules and health systems in a language different to their native language. Findings show a correlation between low Polish language proficiency and likelihood of having children who were not fully vaccinated. The language barrier is part of wider challenges around navigating a new health system. Over a quarter of mothers reported not knowing the Polish vaccine schedule and 17.5% reported not knowing where to get their children vaccinated in Poland.

The findings showed that mothers who were unfamiliar with the Polish healthcare system, specifically those who did not know the vaccination schedule or where to get vaccinated, were likely to vaccinate their children in the next six months. This demonstrates an intention to vaccinate but potential systematic barriers.

An increasing number of studies on vaccine hesitancy consider the context and structural factors influencing vaccine uptake, and the concept of vaccine hesitancy has become widely used as a means to explore the refusal of vaccinations. Contextual influences include historic, social, cultural, environmental, economic, political, and institutional factors, which can all shape vaccine sentiments (i.e., conspiracy theories, religious fatalism, violation of human rights). Individual and group influences include personal perceptions or beliefs of the vaccines and influences from the social environment (i.e., vaccine safety, low risk/severity of disease, mistrust in health institutions, previous negative experiences). Vaccine and vaccination-specific issues include, i.e., no medical need, access, financial cost, or lack of recommendation from providers [47,48,49].

Numerous initiatives at the international, national, and local levels have been implemented to address the healthcare needs of displaced Ukrainians, including those related to vaccine-preventable diseases. Following a public health approach, studies conducted among Ukrainian citizens, as well as among stakeholders and workers from institutions involved in supporting displaced Ukrainians across various sectors, assessed the situation and identified areas requiring intervention (e.g., overcoming language barriers or addressing the need for primary care physicians to provide migrant-sensitive healthcare). Subsequently, appropriate measures aimed to build trust in vaccination, enhance vaccine literacy and improve access to vaccinations in the host country were implemented. Examples of such initiatives include international programmes like AcToVax4NAM and locally implemented projects such as Say YES to Vaccination [12,19,50].

A number of mothers expressed a preference to vaccinate their children back in Ukraine (13.6%). A mother’s intention to return to Ukraine was significantly associated with vaccination intentions in Poland compared with those who were planning to stay. The delay of vaccination can have public health implications for the wider community. Measles and polio outbreaks have been reported in Ukraine since the full-scale invasion, which demonstrates existing critical gaps in coverage [24,51].

Past parental experiences around vaccination, both in their home and host countries, may influence their decisions regarding vaccinating their children [9,43]. When developing intervention strategies to boost vaccination rates, it is essential to address and thoughtfully consider parental concerns rather than overlook them [43].

### 4.4. Limitations of the Study

Our study contributes valuable insights into the factors influencing the perceptions and intentions of mothers to vaccinate their children as they navigate life in a new host country. These insights are essential for designing effective multisectoral public health interventions.

Despite this contribution to the evidence base, we acknowledge some limitations. Due to the convenience sampling method used, and the study’s limited geographic scope (limited to several cities in Poland), participants included in the study are not a representative sample of all Ukrainian mothers in Poland. Given the ongoing uncertainty and mobility of displaced Ukrainians, it was difficult to estimate the number of mothers who resided in Poland with at least one child under the age of 7. Nonetheless, available statistics from Statistics Poland indicate that 417,354 Ukrainian women aged 18–65 were in Poland at the end of 2023 [52]. The data were collected through self-reporting, which may be prone to bias. Data on the child’s vaccination status and compliance with the PNIP were not verified against the child’s vaccination history or vaccination record. This lack of verification should be taken into account when interpreting the results of our study. Our survey was conducted exclusively online, meaning that only individuals with internet access could participate, while those without digital access were excluded. Therefore, our future research efforts should also focus on individuals who are or may be at risk of digital exclusion. Furthermore, online surveys have several limitations, including limited generalizability due to non-random sampling, risks of survey fraud, and participant disinterest, and potential biases introduced, i.e., by the survey administration mode. Given this was a cross-sectional study, it is not possible to determine any causal relationships. Although we analysed many factors that could influence mothers’ intentions to vaccinate their children, these were not all the potential factors that might have an impact. Finally, because intention to vaccinate is deeply context dependent, our findings may be limited to Ukrainians in Poland.

Despite these limitations, we believe the work could be useful for other countries hosting displaced communities. By analysing challenges and motivators surrounding vaccination among Ukrainian communities in their host country, this study aims to inform future efforts to develop targeted vaccination strategies.

## 5. Recommendations and Conclusions

Our findings give an insight into the complex factors at play for Ukrainian mothers in Poland and provide some key considerations for public health programmes.

Efforts to strengthen trust can be developed through effective engagement with these communities. Restoring trust in the healthcare system, public health organisations, vaccines, and vaccine providers requires transparent communication on vaccine risks and benefits and ensures access to credible, science-based information for the public, healthcare professionals, and policymakers. Moving beyond simple information dissemination, the building of meaningful relationships between the health system and Ukrainian refugees will continue to be important. This includes investment in a respectful, culturally contextualized, two-way dialogue with Ukrainian mothers to understand concerns and address questions around vaccination.

Within community engagement efforts, communications strategies should include efforts to address specific concerns around safety. This includes focusing on the particularities of individual concerns rather than overall response rates at the population level. These strategies should be dynamic, and ongoing, to adapt to changing circumstances for displaced mothers and evolving sentiments. Reliable information must be easily accessible in appropriate languages, with careful instructions in how to navigate vaccination schedules between countries. Information campaigns could be launched to communicate scientific facts—using tailored formats like video summaries of scientific articles and plain-language summaries of clinical trials—and to address migrants’ challenges related to vaccines, vaccination, barriers to accessing preventive services, and becoming familiar with the healthcare system in the host country.

Qualitative research with parents and caregivers will allow for deeper investigation into some of the drivers of concern regarding specific vaccine safety and trust in the Polish health system. These insights would be useful in community engagement and communications strategies that focus on vaccination but also on other challenges faced by displaced Ukrainians including school enrolment, social services, and integration. A longitudinal study will allow for analysis into how these dynamics evolve, particularly shifting intentions as duration of displacement is prolonged. Recognizing the unique needs of displaced Ukrainians is crucial when planning vaccination campaigns and incorporating them adequately into the Polish health system and the PNIP to ensure access to life-course vaccinations.

Organisations such as UNICEF and public institutions could further invest in initiatives to strengthen vaccine literacy and guidance on navigating new health systems to increase health equity. Strategies should acknowledge and respond to the environmental, cultural, behavioural, and social factors influencing vaccination decisions. This includes working closely with healthcare workers and healthcare providers to ensure they are equipped with the necessary tools to address concerns empathetically and effectively. By fostering trust through transparent dialogue and community-driven initiatives, health authorities can create a supportive environment where accurate information is not only accessible but also resonates with communities.

## Figures and Tables

**Table 1:** Basic characteristics of the sample according to current vaccination status (N = 2752).

	N (%)	To Date Child Vaccination *				Chi-Sq *p*
		None N-1336	Some N-439	All N-811	Not Sure N-166	
Level of education						
Primary	36 (1.3)	1.7	0.0	0.7	1.8	
Secondary	375 (13.6)	15.5	13.7	10.5	13.9	25.886
Vocational	511 (18.6)	19.6	18.2	17.0	18.7	0.011
Incomplete higher	344 (12.5)	11.2	11.2	14.9	15.1	
Higher/scientific degree	1486 (54.0)	52.0	56.0	56.8	50.6	
Polish language proficiency						
High	145 (5.3)	3.2	6.2	8.4	4.2	
Medium	1039 (37.8)	35.9	39.4	41.3	30.7	65.777
Low	1333 (48.4)	50.6	48.7	44.5	49.4	<0.001
Do not speak Polish at all	214 (7.8)	9.5	5.2	5.2	13.3	
Prefer not to say	21 (0.8)	0.7	0.5	0.6	2.4	
Intention to return to Ukraine *						
As soon as possible	402 (18.3)	20.2	18.1	16.3	13.9	
Probably return soon	453 (20.6)	23.0	18.9	17.8	21.3	30.355
Probably not return even if possible	382 (17.4)	16.7	18.6	17.5	18.9	0.002
Will not return in any case	178 (8.1)	5.9	9.0	11.3	4.9	
Not sure	785 (35.7)	34.2	35.3	37.1	41.0	
Importance of child vaccination						
Low (0–6)	742 (27.0)	36.2	23.5	13.7	27.1	145.267
Average (7–8)	308 (11.2)	11.4	11.8	10.1	13.3	<0.001
High (9–10)	1702 (61.8)	52.5	64.7	76.2	59.6	
Youngest child age						
0–2	984 (35.8)	17.8	49.9	61.0	19.3	470.799
3–4	750 (27.3)	34.7	21.9	16.6	33.7	<0.001
5–7	1018 (37.0)	47.5	28.2	22.3	47.0	

* For 552, there was a lack of systematic data.

**Table 2:** Reasons for Previous Non-Vaccination of the Child by Vaccination Status (N = 1775) *.

Reason for Decision Not to Vaccinate Child Before	Total n (%)	To Date Vaccination (%)		Chi-Sq *p*
		None	Some	
I’m concerned about the safety and side effects of vaccine	382 (21.5)	22.2	19.6	1.288 0.256
I don’t have easy access to healthcare services in Poland	114 (6.4)	7.9	2.1	18.553 <0.001
I don’t know the vaccine schedule/requirements in Poland	495 (27.9)	33.0	12.3	70.460 <0.001
I don’t know where to get vaccinated in Poland	310 (17.5)	20.7	7.5	40.041 <0.001
I don’t trust the healthcare system and/or medical authorities in Poland	109 (6.1)	7.0	3.6	6.305 0.012
I don’t believe my child needs to be vaccinated (don’t believe on the threat of the diseases)	66 (3.7)	4.0	2.7	1.580 0.209
I was advised by health provider not to receive the vaccines (contraindications, etc.)	87 (4.9)	4.8	5.2	0.143 0.706
I refuse to vaccinate my child due to my religious beliefs	25 (1.4)	1.6	0.7	2.208 0.137
I prefer to vaccinate my child when back to Ukraine	241 (13.6)	16.1	5.9	29.127 <0.001
Other	301 (17.0)	14.3	25.1	27.169 <0.001
Difficult to answer/Not sure	228 (12.8)	8.9	24.8	74.823 <0.001

* Mothers declaring full vaccination of their child did not provide reasons.

**Table 3:** Intentions to vaccinate the child in the upcoming months based on reasons for previous non-vaccination according to the Polish vaccination schedule (PNIP) (N = 1480).

Reason for Decision Not to Vaccinate Child Before	Intention to Vaccinate Child in Next 6 Months *			*p*
	Yes	No	Not Sure	
I’m concerned about the safety and side effects of vaccine	23.5	52.9	23.6	82.231 <0.001
I don’t have easy access to healthcare services in Poland	39.6	26.7	33.7	4.523 0.104
I don’t know the vaccine schedule/requirements in Poland	46.3	16.4	37.3	98.197 <0.001
I don’t know where to get vaccinated in Poland	51.7	15.7	32.5	48.451 <0.001
I don’t trust the healthcare system and/or medical authorities in Poland	14.3	62.2	23.5	45.941 <0.001
I don’t believe my child needs to be vaccinated (don’t believe on the threat of the diseases)	7.1	83.9	8.9	68.181 <0.001
I was advised by health provider not to receive the vaccines (contraindications etc.)	32.0	46.7	21.3	6.635 0.036
I refuse to vaccinate my child due to my religious beliefs	-	94.4	5.6	31.093 <0.001
I prefer to vaccinate my child when back to Ukraine	20.0	50.5	29.5	54.579 <0.001
Other	46.2	35.7	18.1	9.300 0.010
Difficult to answer/Not sure	57.7	21.2	21.2	24.015 <0.001

* The distribution of intentions to vaccinate the child when a given reason was not selected was omitted in the table.

**Table 4:** Determinants of intention to get a child vaccinated in Poland in the next 6 months—results of the hierarchical logistic regression estimated in case of non-compliance with the Polish vaccination schedule (PNIP).

	Model 1		Model 2		Model 3	
	*p*	OR (95% CI)	*p*	OR (95% CI)	*p*	OR (95% CI)
Age of the youngest child (cont.)	<0.001	0.85 (0.79–0.91)	<0.001	0.83 (0.76–0.89)	<0.001	0.82 (0.76–0.89)
Compliance with PNIP						
Some	<0.001	2.13 (1.53–2.97)	<0.001	2.22 (1.54–3.18)	<0.001	1.91 (1.33–2.75)
None (ref.)		1.00		1.00		1.00
Return to Ukraine permanently						
As soon as possible	<0.001	0.41 (0.25–0.68)	0.002	0.42 (0.25–0.73)	<0.001	0.37 (0.21–0.65)
Probably as soon as possible	0.460	0.83 (0.51–1.36)	0.464	0.82 (0.49–1.39)	0.246	0.72 (0.42–1.25)
Probably won’t return, even if possible	0.208	1.38 (0.84–2.29)	0.243	1.38 (0.81–2.45)	0.520	1.20 (0.69–2.10)
Will not return in any case (ref.)		1.00		1.00		1.00
I’m concerned about the safety and side effects of vaccines *	-	-	<0.001	0.35 (0.23–0.52)	-	-
I don’t know where to get vaccinated in Poland *	-	-	<0.001	1.84 (1.29–2.64)	0.003	1.74 (1.21–2.50)
I don’t trust the healthcare system and/or medical authorities in Poland *	-	-	0.002	0.30 (0.14–0.64)	0.014	0.39 (0.19–0.84)
I don’t believe my child needs to be vaccinated *	-	-	0.003	0.10 (0.02–0.46)	-	-
I prefer to vaccinate my child when back to Ukraine *	-	-	<0.001	0.43 (0.27–0.69)	<0.001	0.46 (0.28–0.74)
I think it’s important to vaccinate my child (cont.).	-	-			<0.001	1.24 (1.17–1.30)
Constant	0.162	1.458	0.013	2.116	0.005	0.372
R-sq Naglkerke	0.136		0.266		0.300	

* Reference group: no indication of the given reason.

## Data Availability

Data are contained within the article.

## Supplementary Materials

The following supporting information can be downloaded at: https://www.mdpi.com/article/10.3390/vaccines13030325/s1, Study questionnaire; Table S1: PCA for Vaccination Barriers Excluding ’Others’ and ’Difficult to Say’ Options; Table S2: Basic characteristics of the sample according to intention to vaccinate the child.

## Abbreviations

The following abbreviations are used in this manuscript:

PNIP	Polish National Immunisation Programme
BeSD	Behavioural and Social Drivers of vaccination
WHO	World Health Organisation
UNICEF	United Nations International Children’s Emergency Fund
