# Supplementary material for: Determinants of Ukrainian Mothers’ Intentions to Vaccinate Their Children in Poland: A Cross-Sectional Study
Source: Vaccines (Basel). 2025 Mar 19;13(3):325. doi: 10.3390/vaccines13030325 (PMC11945471; doi:10.3390/vaccines13030325)
Supplement: Supplementary file 1 [file vaccines-13-00325-s001.zip › vaccines-3501064-supplementary.pdf]

Supplementary Materials

Supplementary Material_1 Study questionnaire

Ohter #	code	UA	ENG
**1**		**Якою мовою Ви хотіли б пройти опитування?**<br>1; Українська<br>2; Російська	
**2**	**Dobl3a**	**В якій країні Ви зараз проживаєте?**<br>1; Німеччина →→→ кінець інтерв'ю<br>2; Чехія →→→ кінець інтерв'ю<br>**3; Польща**<br>4; Ірландія →→→ кінець інтерв'ю<br>5; Словаччина →→→ кінець інтерв'ю<br>6; Інша →→→ кінець інтерв'ю<br>99; Немає відповіді →→→ кінець інтерв'ю	**In which country do you currently live?**<br>Germany→→→ finish<br>Czech Republic→→→ finish<br>**Poland**<br>Ireland→→→ finish<br>Slovakia→→→ finish<br>Another one→→→ finish<br>No answer→→→ finish
**3**	**Dsex1**	**Вкажіть Вашу стать**<br>1; Чоловік →→→ кінець інтерв'ю<br>2; Жінка	**Please indicate your gender**<br>Male→→→ finish<br>Female
**4**	**Dkid07**	**Чи проживають разом з Вами Ваші діти до 7 років включно?**<br>1; Так<br>**2;** Ні →→→ кінець інтерв'ю	**Do your children under the age of 7 live with you?**<br>Yes<br>No →→→ finish
**5**	**Dkid07a**	**Скільки років Вашій наймолодшій дитині?**<br><br>1; Менше 1 року<br>2; 1 рік<br>3; 2 роки<br>4; 3 роки<br>5; 4 роки<br>6; 5 років<br>7; 6 років<br>8; 7 років	**How old is your youngest child?**<br><br>1. Less than 1 year old<br>2. 1 year old<br>3. 2 years old<br>4. 3 years old<br>5. 4 years old<br>6. 5 years<br>7. 6 years<br>8. 7 years
**6**	**Dage1**	**Скільки Вам повних років?**	**What is your age?**
**7**	**Dedu1**	**Вкажіть рівень Вашої освіти**<br><br>1; Початкова освіта<br>2; Повна середня освіта<br>3; Повна професійно-технічна освіта<br>4; Неповна вища освіта<br>5; Повна вища освіта/ науковий ступінь	**What is your level of education?**<br><br>1. Primary education<br>2. Completed secondary education<br>3. Completed vocational/technical<br>4. Incomplete higher education<br>5. Higher / scientific degree
**8**	**PLjob2**	**Чи працюєте Ви зараз у Польщі?**<br><br>1; Так	**Do you currently work in Poland?**

		2; Ні<br>99; Не хочу відповідати	1. Yes<br>2. No<br>99. I prefer not to say
**9**	**DPLobl1**<br><br>**List**	**В якому воєводстві Польщі Ви проживаєте?**<br>1; Мазовецьке (центр Варшава)<br>2; Нижньосілезьке (Вроцлав)<br>3; Куявсько-Поморське (Бидгош, Торунь)<br>4; Люблінське (Люблін)<br>5; Любуське (Гожув, Зелена Гура)<br>6; Лодзинське (Лодзь)<br>7; Малопольське (Краків)<br>8; Опольське (Ополе)<br>9; Підкарпатське (Жешув)<br>10; Підляське (Білосток)<br>11; Поморське (Ґданськ)<br>12; Свентокшиське (Кельці)<br>13; Сілезьке (Катовиці)<br>14; Великопольське (Познань)<br>15; Вармінсько-Мазурське (Ольштин)<br>16; Західнопоморське (Щецин)	**Where in Poland do you currently live?**<br>**Drop down: List of provinces**<br>1. Masovia<br>2. Lower Silesia<br>3. Kuyavia-Pomerania<br>4. Lublin<br>5. Lubusz<br>6. Łódź<br>7. Lesser Poland<br>8. Opole<br>9. Subcarpathia<br>10. Podlasie<br>11. Pomerania<br>12. Świętokrzyskie Voivodeship<br>13. Silesia<br>14. Greater Poland<br>15. Warmia-Masuria<br>16. West Pomerania
**10**	**Lang2Pl**	**Як Ви можете оцінити свій рівень володіння польською мовою?**<br><br>1; Високий<br>2; Середній<br>3; Низький<br>4; Не володію польською мовою взагалі<br>99; Не хочу відповідати	**How would you assess your level of Polish language proficiency?**<br><br>1. High<br>2. Medium<br>3. Low<br>4. I do not speak Polish at all<br>99. I prefer not to say
**11**	**Dobl5**<br><br>**List**	**В якій області України Ви проживали до 24 лютого 2022?**<br>1; Вінницька<br>2; Волинська<br>3; Дніпропетровська<br>4; Донецька<br>5; Житомирська<br>6; Закарпатська<br>7; Запорізька<br>8; Івано-Франківська<br>9; Київська<br>25; м. Київ<br>10; Кіровоградська<br>11; Луганська<br>12; Львівська<br>13; Миколаївська<br>14; Одеська<br>15; Полтавська<br>16; Рівненська	**Drop down: List of oblasts**<br><br>1. Vinnytsia<br>2. Volyn<br>3. Dnipropetrovsk<br>4. Donetsk<br>5. Zhytomyr<br>6. Zakarpattia<br>7. Zaporizhzhia<br>8. Ivano-Frankivsk<br>9. Kyiv oblast<br>10. Kirovohrad<br>11. Luhansk<br>12. Lviv<br>13. Mykolaiv<br>14. Odesa<br>15. Poltava<br>16. Rivne<br>17. Sumy

		17; Сумська<br>18; Тернопільська<br>19; Харківська<br>20; Херсонська<br>21; Хмельницька<br>22; Черкаська<br>23; Чернівецька<br>24; Чернігівська<br>99; Немає відповіді	18. Ternopil<br>19. Kharkiv<br>20. Kherson<br>21. Khmelnytskyi<br>22. Cherkasy<br>23. Chernivtsi<br>24. Chernihiv<br>25. Kyiv
**12**	**PL07Un1**	**Чи погоджуєтеся Ви з твердженням про те, що вакцинувати свою дитину важливо?**<br><br>1; 0: Зовсім не погоджуюся<br>2; 1<br>3; 2<br>4; 3<br>5; 4<br>6; 5<br>7; 6<br>8; 7<br>9; 8<br>10; 9<br>11; 10: Повністю погоджуюся	**I think it's important to vaccinate my child**<br><br>0: I strongly disagree<br>1<br>2<br>3<br>4<br>5<br>6<br>7<br>8<br>9<br>10: I strongly agree
**13**	**PL07Un2**	**Як Ви думаєте, більшість Вашої родини та друзів хочуть, щоб Ваша дитина була вакцинована?**<br>1; Так<br>2; Ні<br>99; Важко відповісти	**Do you think most of your close family and friends want you to get your child vaccinated?**<br>1. No<br>2. Yes<br>99. Difficult to answer/Not sure
**14**	**PL07Un3**	**Чи знаєте Ви, як зробити щеплення своїй дитині в Польщі?**<br><br>1; Так<br>2; Ні	**Do you know how to get your child vaccinated in Poland?**<br><br>1. No<br>2. Yes
**15**	**PL07Un4**	**У Польщі є календар вакцинації дітей. Чи отримувала ваша дитина щеплення згідно цього календаря?**<br><br>1; Ні, не отримувала жодних щеплень<br>2; Отримувала деякі щеплення<br>3; Отримала усі щеплення<br>99; Важко відповісти	**Poland has a schedule of vaccines for children. As far as you know, has your child received none, some, or all of these vaccines?**<br>1. None<br>2. Some<br>3. All<br>99. Difficult to answer/Not sure
**16**	**PL07Un5**<br><br>Серед тих, хто	**Яка причина вашого рішення не вакцинувати Вашу дитину?**<br>Можливо ДЕКІЛЬКА відповідей<br><br>1; Я стурбована безпечністю та побічними ефектами від вакцин	If some or none: **what was the reason for your decision not to vaccinate your child**?<br>MULTIPLE RESPONSE<br><br>1. I'm concerned about the safety and side effects

	PL07Un4=1 або 2<br><br>**Множинне**	2; Я не маю доступу до медичних послуг у Польщі<br>3; Я не знаю графіку вакцинації/вимог в Польщі<br>4; Я не знаю, де зробити щеплення в Польщі<br>5; Я не довіряю системі охорони здоров'я та/або медичним органам у Польщі<br>6; Я не думаю, що моїй дитині потрібно робити щеплення (не вірю в загрозу захворювань)<br>7; Лікар порадив мені не робити щеплення (протипоказання тощо)<br>8; Я відмовляюся робити щеплення своїй дитині через свої релігійні переконання<br>9; Я вважаю за краще вакцинувати свою дитину, коли повернуся в Україну<br>97; Інше (вкажіть)______<br>99; Важко відповісти	of vaccines<br>2. I don't have easy access to healthcare services in Poland<br>3. I don't know the vaccine schedule/requirements in Poland<br>4. I don't know where to get vaccinated in Poland<br>5. I don't trust the healthcare system and/or medical authorities in Poland<br>6. I don't believe my child needs to be vaccinated (don't believe on the threat of the diseases)<br>7. I was advised by health provider not to receive the vaccines (contraindications, etc)<br>8. I refuse to vaccinate my child due to my religious beliefs<br>9. I prefer to vaccinate my child when back to Ukraine<br>97. Other (please, specify)<br>99. Difficult to answer/Not sure
**17**	**PL07Un6**	**Наскільки Ви довіряєте медичним працівникам, які проводять вакцинацію дітей?**<br><br>1; Зовсім не довіряю<br>2; Не дуже довіряю<br>3; Переважно довіряю<br>4; Повністю довіряю<br>99; Важко відповісти	**How much do you trust the healthcare providers who vaccinate children?**<br><br>1. Do not trust<br>2. Somewhat trust<br>3. Mostly trust<br>4. Fully trust<br>99. Difficult to answer/Not sure
**18**	**PL07Un7**<br><br>**Множинне**	**Що могли б зробити медичні працівники, щоб краще відповісти на ваші запитання щодо вакцинації?**<br>До ДВОХ відповідей<br><br>1; Надавати інформацію рідною для мене мовою<br>2; Збільшити час консультації<br>3; Врахувати попередні щеплення моєї дитини<br>97; Інше (вкажіть) _______<br>99; Важко відповісти	**What should vaccine providers do to better address your concerns, if anything?** UP TO TWO ANSWERS<br><br>1. Provide the information in my language<br>2. Spend more time during the consultations<br>3. Review my child vaccine records<br>97. Other (specify)<br>99. Difficult to answer/Not sure
**19**	**PL07Un8**	**Чи є у Вас паспорт (довідка) вакцинації або будь-які записи про щеплення Ваших дітей, і чи брали Ви їх, виїжджаючи з України?**<br><br>1; Так, маю (копія або оригінальний документ)	**Do you have a vaccination passport (certificate) or any vaccination records of your children, and did you take it with you when leaving Ukraine?**<br><br>1. Yes, I have it (a copy or actual record)<br>2. No, I don't have it (and I can't access it)<br>99. I prefer not to answer

		2; Ні, не маю (і не можу отримати до них доступ)<br>99. Не хочу відповідати	
**20**	**PL07Un9**	**Чи виникали у Вас проблеми у Польщі з розумінням або перекладом Ваших записів про щеплення?**<br><br>1; Так<br>2; Ні<br>99; Важко відповісти	**Did you have any issues with understanding or translating your vaccine records?**<br><br>1. Yes<br>2. No<br>99. I don't know
**21**	**PL07Un10**	**Чи маєте Ви медичне страхування в Польщі (державна або приватна)?**<br><br>1; Так<br>2; Ні<br>99; Важко відповісти	**Do you have any kind of health care coverage in Poland (health insurance, private health insurance, etc)?**<br><br>1. Yes<br>2. No<br>99. I don't know
**22**	**PL07Un11**	**Чи стикалися Ви з будь-якими труднощами, коли намагались зробити щеплення у Польщі?**<br><br>1; Не намагались зробити щеплення<br>2; Ні, не стикались з труднощами<br>3; Так (опишіть, які саме проблеми) ________	**Have you encountered barriers when you tried to access vaccination services in Poland?**<br><br>1. I haven't tried to access<br>2. No, we did not face any difficulties<br>3. Yes (please, describe) ________
**23**	**PL07Un12**	**Чи маєте Ви намір вакцинувати свою дитину протягом наступних 6 місяців у Польщі?**<br><br>1; Так<br>2; Ні<br>99; Важко відповісти	**Do you intend to vaccinate your child in the next 6 months in Poland?**<br><br>1. Yes<br>2. No<br>99. Difficult to answer/Not sure
**24**	**PL07Un13**<br><br>**Множинне**	**Яким джерелам інформації про вакцинацію ви довіряєте найбільше?** Можливі декілька відповідей<br><br>1; Офіційні медичні установи<br>2; Телебачення<br>3; Радіо<br>4; Газети<br>5; Соціальні мережі<br>6; YouTube<br>7; Батьки інших дітей, що живуть у Польщі<br>1Лікарі<br>8; Родина<br>9; Друзі<br>97; Інше (вкажіть) ________<br>98; Жодному не довіряю<br>99; Важко відповісти	**Which source of vaccine information do you trust the most?** MULTIPLE RESPONSE<br><br>1. Official health institutes<br>2. TV<br>3. Radio<br>4. Newspapers<br>5. Social media<br>6. YouTube<br>7. Other parents from Ukraine living here<br>8. Doctors<br>9. Family<br>10. Friends<br>97. Other (specify)<br>98. I don't trust any of them<br>99. Difficult to answer / I prefer not to say

**25**	**PL07U n14**<br><br>**Множинне**	**Хто з переліку найбільше впливає на Ваше рішення вакцинувати або не вакцинувати дитину?**<br>Можливі декілька відповідей<br><br>1; Родина/сім'я<br>2; Друзі<br>3; Батьки інших дітей, що живуть у Польщі<br>4; Релігійні лідери<br>5; Колеги по роботі<br>6; Школа<br>7; Лікарі або інші медичні працівники<br>8; Знаменитості або інші впливові особистості<br>9; Уповноважені працівники установ з охорони здоров'я<br>97; Інше (вкажіть) ________<br>98; Ніхто з перелічених<br>99; Важко відповісти	**Which of the following has the greatest influence on your decision on whether to vaccinate your child, or NOT?** MULTIPLE RESPONSE<br><br>1. Family<br>2. Friends<br>3. Other parents<br>4. Religious leaders<br>5. Coworkers<br>6. School<br>7. Doctors or other healthcare workers<br>8. Celebrities or other influencers<br>9. Public health figures<br>97.Other (specify)<br>98. None<br>99. Difficult to answer/Not sure
**26**		Анкета майже завершилась, залишилось ще декілька питань.	The questionnaire is almost finished, there are still a few questions left.
**27**	**PL07U n15**	**Коли вашій дитині потрібна медична допомога, куди Ви звертаєтесь?**<br><br>1; До польського лікаря у Польщі<br>2; До українського лікаря у Польщі<br>3; До лікаря в Україні<br>4; Не маю доступу до медичних послуг<br>99; Важко відповісти	**When your child needs medical care, where do you go?**<br><br>1. To a non-Ukrainian doctor in Poland<br>2. To a Ukrainian doctor in Poland<br>3. To a doctor in Ukraine<br>4. I can't access health care<br>99. I don't know
**28**	**PL07U n16**	**Що з наведеного найкраще описує ваше фінансове становище?**<br><br>1; Недостатньо коштів<br>2; Достатньо лише на повсякденні витрати<br>3; В основному достатньо<br>4; Достатньо на майже все, що потрібно<br>5; Більш ніж достатньо<br>99; Не хочу відповідати	**Which of the following best describes your financial situation?**<br><br>1. Not enough<br>2. Enough, but only for everyday expenses<br>3. Mostly enough<br>4. Enough for almost everything<br>5. More than enough<br>99. I prefer not to answer
**29**	**PL07U n17**	**Чи плануєте Ви повертатися в Україну на постійне проживання?**<br><br>1; Однозначно повернуся в найближчий час<br>2; Можливо повернуся в найближчий час	**How likely are you to return to Ukraine permanently?**<br><br>1. I will return to Ukraine as soon as possible<br>2. I'll probably return as soon as possible<br>3. I probably WON'T return, even if it's

		3; Скоріше НЕ повернуся, навіть якщо це буде можливо<br>4; НЕ планую повертатися взагалі<br>99; Важко відповісти	possible<br>4. I will not return in any case<br>99. Difficult to answer/Not sure
**30**	**PL07Un18**	**У вас є номер PESEL?**<br>1; Так<br>2; Ні<br>99; Важко відповісти	**Do you have a PESEL number?**<br><br>1. Yes<br>2. No<br>99. I prefer not to answer
**ВИПАДКОВИЙ ВИБІР БЛОКУ. INTERVENTION**			
**31**	**PLUn27TRUST**	**Хочемо Вас поінформувати про наступне:**<br><br>Запишіть своїх дітей на планові щеплення, поки ви перебуваєте у Польщі! Захистіть їх здоров'я та вбережіть від захворювань, яким можна запобігти за допомогою вакцин.<br>Система охорони здоров’я в Польщі має потужну програму імунізації, яка успішно запобігає серйозним захворюванням у дітей за допомогою найякісніших вакцин.<br><br>1;Зрозуміло	**We would like to inform you about the following**<br><br>*Schedule routine vaccinations for your children while you are in Poland! Protect their health and shield them from vaccine preventable diseases.*<br>*Polish health system has a strong immunization program, that has been successful in preventing serious diseases in children using the highest quality vaccines.*<br><br>Understandably
**32**	**PLUn27ACCESS**	**Хочемо Вас поінформувати про наступне:**<br><br>Запишіть своїх дітей на планові щеплення, поки ви перебуваєте в Польщі! Захистіть їх здоров'я та вбережіть від захворювань, яким можна запобігти за допомогою вакцин.<br>Зробити щеплення дитині в Польщі – це легко і зручно.<br><br>1;Зрозуміло	**We would like to inform you about the following**<br>*Schedule routine vaccinations for your children while you are in Poland! Protect their health and shield them from preventable diseases.*<br>*Getting your child vaccinated in Poland is easy and convenient.*<br><br>Understandably
**33**	**PLUn27RISK**	**Хочемо Вас поінформувати про наступне:**<br><br>Запишіть своїх дітей на планові щеплення, поки ви перебуваєте в Польщі! Захистіть їх здоров'я та вбережіть від захворювань, яким можна запобігти за допомогою вакцин.<br>Вакцинація вашої дитини під час війни важливіша, ніж будь-коли, оскільки в цей час діти можуть бути більш вразливі до інфекцій.<br><br>1;Зрозуміло	**We would like to inform you about the following**<br>*Schedule routine vaccinations for your children while you are in Poland! Protect their health and shield them from preventable diseases.*<br>*Vaccinating your child during emergency is more important than ever as your child might be more vulnerable to infections.*<br><br>Understandably

**34**	**PLUn27CONTROL**<br>(питання без варіантів відповідей, звичайний текст ставитесь в блок разом з наступними)	Наважтеся бути здоровими.	*Dare to be healthy.*
**Задаються всім**			
**35**	**PL07Un28**	**Чи погоджуєтеся Ви з тим, що вакцинувати свою дитину важливо?**<br><br>1; 0: Зовсім не погоджуюся<br>2; 1<br>3; 2<br>4; 3<br>5; 4<br>6; 5<br>7; 6<br>8; 7<br>9; 8<br>10; 9<br>11; 10: Повністю погоджуюся	**I think it's important to vaccinate my child**<br><br>0: I strongly disagree<br>1<br>2<br>3<br>4<br>5<br>6<br>7<br>8<br>9<br>10: I strongly agree
**36**	**PL07Un29**	**Чи маєте Ви намір вакцинувати свою дитину протягом наступних 6 місяців у Польщі?**<br>1; Так<br>2; Ні<br>99; Важко відповісти	**Do you intend to vaccinate your child in the next 6 months in Poland?**<br><br>1. Yes<br>2. No<br>99. Difficult to answer/Not sure
**37**	**PL07Un31**	Натисніть на посилання нижче та зареєструйтеся на прийом для вакцинації Вашої дитини.<br>1;ЗАПИСАТИСЬ НА ПРИЙОМ ДО ЛІКАРЯ (перехід на сайт)<br>2;Відмовитись (фінальна сторінка)	*Click below and make an appointment to get your child vaccinated.*<br><br>**MAKE AN APPOINTMENT WITH A DOCTOR**<br><br>**Refuse**

Table S1. PCA for Vaccination Barriers Excluding 'Others' and 'Difficult to Say' Options

Rotated Component Matrix [a]	Principal Component		
	1	2	3
a) I'm concerned about the safety and side effects of vaccines		.537	
b) I don't have easy access to healthcare services in Poland	.477		
c) I don't know the vaccine schedule/requirements in Poland	.770		
d) I don't know where to get vaccinated in Poland	.809		
e) I don't trust the healthcare system and/or medical authorities in Poland			.626
f) I don't believe my child needs to be vaccinated (don't believe on the threat of the diseases)		.725	
g) I was advised by health provider not to receive the vaccines (contraindications, etc.)			
h) I refuse to vaccinate my child due to my religious beliefs		.692	
i) I prefer to vaccinate my child when back to Ukraine			.794

Factor Extraction Method – Principal Component Analysis.
Rotation Method – Varimax with Kaiser Normalization
a. Rotation converged in 4 iterations

Table S2. Basic characteristics of the sample according to intention to vaccinate the child (N=2346).

	Intention to vaccinate			Chi-sq
	Yes	No	Not sure	p
Level of education				
Primary	1.4	1.2	1.2	
Secondary	13.1	12.6	16.6	16.242
Vocational	17.5	19.9	18.0	0.039
Incomplete higher	14.1	9.1	12.1	
Higher/scientific degree	54.0	57.2	52.1	
Polish language proficiency				

High	6.6	4.8	2.5	
Medium	37.6	37.4	37.4	26.274
Low	49.1	46.9	51.3	<0.001
Do not speak Polish at all	6.2	10.4	7.6	
Prefer not to say	0.5	0.4	1.2	
Intention to return to Ukraine				
As soon as possible	13.7	27.1	15.6	
Probably return soon	19.5	22.0	21.1	102.598
Probably not return even if possible	22.2	12.9	12.6	<0.001
Will not return in any case	9.2	8.6	4.6	
Not sure	35.5	29.4	46.2	
Importance of child vaccination				
Low (0-6)	10.6	42.0	31.5	266.889
Average (7-8)	10.7	10.8	13.7	<0.001
High (9-10)	78.7	47.2	54.8	
Youngest child age				
0-2 y.	52.8	24.3	17.6	254.824
3-4 y.	18.6	34.1	36.2	<0.001
5-7 y.	28.6	41.6	46.2	
